# Potential role of weather, soil and plant microbial communities in rapid decline of apple trees

**DOI:** 10.1371/journal.pone.0213293

**Published:** 2019-03-06

**Authors:** Jugpreet Singh, Katchen Julliany Pereira Silva, Marc Fuchs, Awais Khan

**Affiliations:** Plant Pathology and Plant-Microbe Biology Section, Cornell University, Geneva, NY, United Sttaes of America; Estacion Experimental del Zaidin, SPAIN

## Abstract

An unusual decline and collapse of young established trees known as “rapid apple decline” (RAD) has become a major concern for apple growers, particularly in the northeastern United States. This decline is characterized by stunted growth, pale yellow to reddish leaves, and tree collapse within weeks after onset of symptoms. We studied declining apple trees to identify potential involvement of abiotic and biotic stresses. We used 16S and ITS to profile bacterial and fungal communities in the soil, rhizosphere, roots, and shoots and tested for the presence of six viruses in scions and rootstocks of symptomatic and asymptomatic trees. The viruses detected were not associated with RAD symptoms. Bacterial and fungal populations were highly variable in plant tissue, soil and rhizosphere samples, with bacteroidetes, firmicutes, proteobacteria, acidobacteria, and actinobacteria the predominant bacterial classes in various samples. ‘Alphaproteobacteria-rickettsiales’, a bacterial class usually reduced in water-limiting soils, had significantly low abundance in root samples of symptomatic trees. Basidiomycota and Ascomycota fungal classes were the most common fungal classes observed, but neither showed differential enrichment between symptomatic and asymptomatic trees. Analyzing weather data showed an extremely cold winter followed by drought in 2015–2016, which likely weakened the trees to make them more susceptible to varied stresses. In addition, similar physical and nutritional soil composition from symptomatic and asymptomatic trees rules out the role of nutritional stress in RAD. Necrotic lesions and wood decay symptoms dispersing from bark or vascular cambium towards the heartwood were observed primarily below the graft union of declining apple trees, suggesting that the rootstock is the originating point of RAD. We speculate that differences in abiotic factors such as moisture levels in declining roots in combination with extreme weather profiles might cause RAD but cannot clearly rule out the involvement of other factors.

## Introduction

“Rapid apple decline” (RAD) describes a decline and collapse of young apple trees. This decline has become a major concern for growers in apple producing areas in the central, northeastern, and northwestern United States and in Ontario, Canada in recent years [1–4]. A similar situation was first described in north-central Washington State orchards in 1983 [5], and fifteen years later in southern British Columbia, Canada [6]. RAD is usually characterized by stunted tree growth, chlorotic canopy, and tree collapse within weeks after the onset of symptom development [2–4]. Symptoms generally first appear on one limb, with small, rolled leaves and reduced terminal growth, followed by the full tree canopy manifesting pale yellow to reddish leaves. Cankers and shedding are visible at the graft union, and wood necrosis progresses upstream to the trunk of the tree. The root system generally appears healthy, except that small feeder roots are absent [2, 5]. No common root rot pathogens or nutrient deficiency has been associated with RAD. After originating in one point of an orchard, the disorder seems to spread to adjacent trees. Symptomatic trees are usually removed from orchards due to poor productivity; however, if kept in the orchard, the symptoms may spread throughout the tree within a single year and ultimately lead to tree death.

Despite extensive speculation on the potential causes of RAD, the causative agent is still unknown. For example, cultivar-rootstock incompatibility, extreme weather conditions, wood-boring insects, and pathogen infection have all been proposed as possible causes. Trees presenting incompatible grafts are frequently described as displaying breaks or malformations at the graft union, leaf chlorosis, early defoliation, plant wilt, and premature death. Unfavorable weather conditions have also been suggested to be involved in RAD [7–9]. For instance, freezing temperatures can cause direct injury to plant tissue, making them vulnerable to secondary abiotic or biotic stresses [8–9]. Likewise, drought or flooding may potentially cause retarded shoot and leaf growth, leaf chlorosis and defoliation, root necrosis, wilting, and eventually plant senescence [10–16]. Wood-boring insects can also cause serious damage or death of apple trees and have been identified in trees with RAD [17]. Insect infested trees usually have a sickly appearance, a sparse and pale-colored foliage, and can die with a heavy fruit crop during the fruit maturation stage. Although insect borer injury to the graft union or trunk has been reported in RAD-symptomatic areas, it is believed that insects take advantage of already declining trees, and compound the injury by providing an entryway for destructive fungi [2, 4, 17].

The involvement of plant pathogens in RAD is still a matter of speculation. Many important diseases of apple trees are caused by pathogens that initiate infections at wounds caused by insects, humans, machinery, fire, lightning, wind, hail, animals, or nutritional and physiological disorders [18–19]. Symptoms from other microbes such as wood-rotting and *Phytophthora* crown rot pathogens and mycoplasma-like organisms showed partial matches to RAD symptoms [5, 20–22]. In contrast, the necrotic symptoms of the inner bark on RAD-affected trees were more likely to be from latent apple viruses such as apple stem pitting virus and apple stem grooving virus [2, 4]. Although apple luteovirus 1 (*ALV1*) was recently characterized from trees with RAD [23], the association between the presence of *ALV*1 and RAD symptoms is weak. Therefore, no virus has been confirmed as the causative agent for RAD [2]. It might be possible that RAD is caused by an as yet unknown pathogen.

The identification of microorganisms responsible for plant disease has relied mainly on culture-dependent techniques and PCR amplification of genomic DNA [24]. However, these techniques are specific and can miss certain infectious microorganisms or groups of organisms that cause diseases. Microbial communities have been shown to have synergistic effects by improving agronomical features, such as limiting or preventing attacks by phytopathogens [25–27] or causing the establishment and development of plant diseases [20, 24, 27–31]. High throughput sequencing approaches are powerful tools to determine the involvement of known and new organisms in disease etiology and permit the investigation of complex microbiomes [20, 27, 32, 33]. Routine 16S and ITS sequencing allow rapid identification of soil, rhizosphere, or endophyte bacterial and fungal communities, respectively [34–39]. For example, a bacterial community potentially interacting with a colonizing fungus was isolated from the grapevine trunk fungal disease “esca” [33]. Similarly, three fungi typically not considered root pathogens have been reported as causal agents of apple replant disease [20]. A comparative analysis between healthy and declining trees can help identify differentially abundant classes of bacteria and fungus and therefore assist in the identification of their putative role in RAD.

We carried out a study in an orchard with a block of ‘HoneyCrisp’ trees showing symptoms of RAD to identify the potential role of different abiotic and biotic stresses. We characterized the morphological status of wood from RAD symptomatic trees to localize the origin of necrosis, compared microbial community profiles of soil, rhizosphere, roots, and shoots of symptomatic and asymptomatic trees to identify biotic agents involved in RAD, and analyzed weather data and physical characteristics of soil to investigate the involvement of abiotic stresses.

## Materials and methods

### Site description and weather data

The experimental orchard is located in a commercial apple orchard in Newark, Wayne County, New York in the United States (43°7’30”N, 77°5’31”W, and 170m elevation). The orchard has more than 30 cultivars of apples grafted on different rootstocks. The majority of declining trees were observed in a block of ‘HoneyCrisp’. The block had 1,700 ‘HoneyCrisp’ trees grafted onto the Malling 9 (M.9 NIC 29) rootstock. This block was established in 2010, in a high-density planting system, with 1.25m x 4.25m spacing between trees and rows. The soil type is characterized as silty loam and belongs to the hydrologic soil group B/D with moderate to very slow infiltration rate and moderate potential for frost action according to the United States Department of Agriculture (USDA)—Natural Resources Conservation Service Soil Climate Analysis Network (NRCS) (USDA-NRCS, 2017). The water movement in the most restrictive layer and the shrink-swell potential is low in this area. The area is considered moderately well drained, with low probability of flooding and ponding. Annual precipitation in the area varies from 787 to 1,447mm, and the frost-free period varies from 100 to 190 days.

Weather datasets from 2013 to 2017 were accessed from three weather stations located in Phelps, Farmington, and Sodus, New York using the Network for Environment and Weather Applications (NEWA) (http://newa.cornell.edu) [40]. These three locations were within 15 miles of the ‘HoneyCrisp’ apple orchard selected for this study. Weather data included temperature (maximum, average, and minimum), precipitation, relative humidity, leaf wetness, and wind speed. There were no major differences in weather parameters among the three stations. Therefore, datasets from three weather stations were used to calculate monthly mean values for each year.

### Sampling for soil, microbiome and virus analysis

Asymptomatic and symptomatic (declining) apple trees were dispersed across the experimental orchard. Four rows (R1-R4) in the north end of the block had clearly declining as well as healthy-looking trees. The two central rows (R2 and R3) were selected for soil and tree tissue sampling, avoiding outer open rows to minimize experimental error (S1A Fig). Three sets of trees (two asymptomatic trees on either side of a symptomatic tree) were sampled from R2 (S1B Fig). In R3, 20 (10 asymptomatic and 10 symptomatic) trees were randomly selected and sampled (S1C Fig).

**Fig 1:**
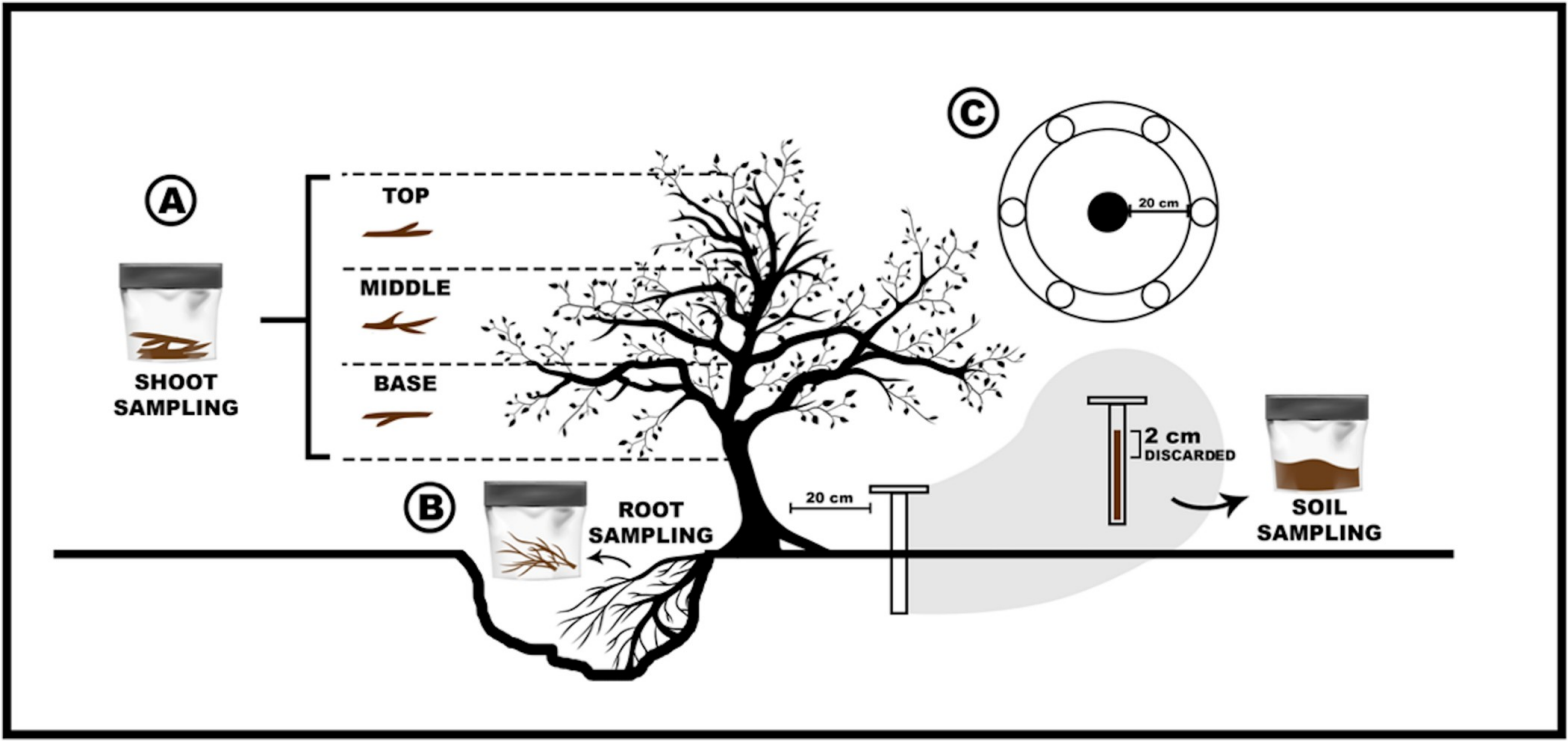
Schematic overview of the sampling strategy of soil and tree samples in a ‘HoneyCrisp’ orchard block with rapid apple decline (RAD). Sampling and pooling of shoots from three different positions in the tree canopy (top, middle, and base) (A), Sampling of root tissue and rhizosphere (B). Soil sampling for the analysis of soil physical and chemical properties (C).

A total of 87 samples were collected from shoots (13 symptomatic, 16 asymptomatic), roots (13 symptomatic, 16 asymptomatic), and the rhizosphere (13 symptomatic, 16 asymptomatic) of ‘HoneyCrisp’ trees selected in R2 and R3. For sampling, shoots (30cm in length) were cut with pruning shears from three different positions in the tree canopy (top, middle, and base) and pooled together (Fig 1A). Soil surrounding the tree rootstock was dug out to expose lateral roots for root and rhizosphere sampling. Five root pieces (5-20cm) adjacent to the rootstock trunk were collected using pruning shears and a chisel (Fig 1B). Pruning shears, shovels, chisels, and augers used during sampling were cleaned with 50% bleach between uses to avoid cross contamination, and gloves were changed between samples.

A total of nine bulk soil samples were collected from six asymptomatic and three symptomatic trees in R2 using a soil auger (4.5 cm Ø). For each sample, six soil cores (~20 cm depth) were collected around the tree, approximately 20cm from the center of the tree trunk. The first 2 cm of each sample was discarded, and the remaining soil was combined, sieved with a 2-mm mesh, and homogenized (Fig 1C). One portion of all soil samples was kept in -80°C for DNA extraction. Another portion of each sample was used for soil physicochemical analysis at the Soil Lab, Cornell University in Ithaca, New York. Plant material and soil samples were kept on ice and carefully transported to the laboratory in a cold container. In the lab, roots were gently shaken inside sampling bags and the rhizosphere soil was carefully detached and transferred to a sterile container. Shoot, root, and rhizosphere samples were processed within 24 hours.

### Visual characterization of rootstocks of symptomatic tress

At the end of the growing season in 2017, eighteen symptomatic apple trees, a subset of the trees that were sampled for microbiome and virus testing, were pulled out from rows R2 and R3. The trunks were cut 30cm above the graft union and brought to the lab for visual characterization of symptoms. Three parallel cuts (5 cm each) were made above and below the graft union to verify the presence of abnormal wood, as well as signs and/or symptoms of potential pathogen and insect damage. High-resolution images of the cross section of each cut piece were taken to analyze necrotic lesions as the percentage of necrotic wood area to the total cross section area. A macro in ImageJ (version 1.51 h - http://imagej.nih.gov/ij) was used to convert pictures to a binary format, assigning white and black colors to healthy and pigmented tissue, respectively. The outputs were used to estimate healthy and pigmented necrotic wood area. The image data were further evaluated to localize the origin and progress of the necrosis using the following scale: DS = 1, external wood discoloration, DS = 2, internal wood discoloration, DS = 3, co-occurrence of external and internal wood discoloration, DS = 4, edge-shaped discoloration (<50%), DS = 5, edge-shaped discoloration (>50%), and DS = 6, circular discoloration.

### Virus detection

Shoot and root samples of asymptomatic and symptomatic trees were tested for the presence of viruses using double-antibody sandwich enzyme-linked immunosorbent assay (DAS-ELISA) and commercial antibodies (Bioreba, Reinach, Switzerland) according to the manufacturer's instructions. Viruses assayed were apple stem grooving virus (*ASGV*), apple stem pitting virus (*ASPV*), apple chlorotic leafspot virus (*ACLSV*), tomato ringspot virus (*ToRSV*), apple mosaic virus (*APMV*), and tobacco ringspot virus (*TRSV*). Briefly, shoot and root samples (0.5g) were homogenized in Bioreba universal extraction bags containing extraction buffer (5ml) using a HOMEX 6 (Bioreba, Reinach, Switzerland). DAS-ELISA was conducted as follow: Specific polyclonal antibodies were diluted 1,000 times in coating buffer, dispensed into wells of a microtiter plate (100 μl/well), and incubated overnight at 4°C. Plates were washed three times with phosphate-buffered saline (PBS) containing 0.05% Tween 20 and 500 μl of sample extracts were added to two wells and kept overnight at 4°C. After three washes with PBS-Tween, 100 μl of conjugate antibody diluted 1,000 times were added and incubated 4h at 30°C. After adding the substrate, absorbance readings were recorded using a microplate reader (EL800, Biotech. Instruments, USA) at 405 nm. Samples were considered virus-positive when the average absorbance values (A_405nm_) were at least three times higher than those of the negative control.

### Sample processing and DNA extraction

Shoot and root samples were processed prior to DNA extraction. Shoots and roots were first washed with sterile water, blotted dry on sterile absorbent paper, and transferred to a laminar flow hood. The bark of the shoots and the cortex of the roots were removed to trim the sample ends. Wood samples were cut horizontally, and small wood chips were quickly collected using a sterile scalpel and transferred to sterile bags. Samples were immediately frozen in liquid nitrogen. Approximately 100 mg of shoot or root tissue were disrupted and homogenized using a tissue lyser II (Qiagen, Crawley, UK). Three 5 mm stainless steel beads were used per sample. Three rounds of 45 s of grinding were performed at a frequency of 30 Hz. Total genomic DNA was extracted from shoots and roots using DNeasy Plant Mini Kit (Qiagen, Germany), and microbial DNA was extracted from bulk soil and rhizosphere samples using the MoBio Power Soil DNA isolation kit (Qiagen, Germany), according to the manufacturer’s instructions. Quality and quantity of extracted DNA was assessed by NanoDrop (absorbance ratio at both 260/280 and 230/260 nm) and by electrophoresis on 1.5% agarose gels. Concentration of extracted DNA was adjusted to 5ng/μl and samples were stored at -80°C until use.

### Characterization of microbial communities

Bacterial and fungal communities were characterized in soil, shoot, root, and rhizosphere samples. The gene‐specific primers V3-357F (5’-CCTACGGGNGGCWGCAG-3’) and V4-805R (5’-GACTACHVGGGTATCTAATCC-3’) targeting the V3 and V4 regions of 16S rRNA gene were used to study bacterial communities (Klindworth et al., 2013; Yim et al., 2015). The gene‐specific primers ITS1F (5’-TCCGTAGGTGAACCTGCGG-3’) and ITS4R (5’-TCCTCCGCTTATTGATATGC-3’), targeting the two transcribed intergenic spacers (ITS) ITS1 and ITS4 rDNA regions were used to study fungal communities (Manter and Vivanco, 2007). The forward and reverse Illumina overhang adapter sequences added to locus‐specific sequences were: 5’-TCGTCGGCAGCGTCAGATGTGTATAAGAGACAG-3’ and 5’-GTCTCGTGGGCTCGGAGATGTGTATAAGAGACAG-3’, respectively. DNA was amplified by PCR in a reaction mixture (25 μl final volume) consisting of 2.5 μl of microbial DNA (5 ng/μl), 5 μl of each amplicon PCR primer (1 μM), and 12.5 μl of 2x KAPA HiFi HotStart ReadyMix (KAPA Biosystems). The PCR amplification conditions for 16S rRNA region were as follows: 95°C for 3 min; 25 cycles of denaturation at 95°C for 30 s; hybridization at 55°C for 30 s; extension at 72°C for 30 s; and a final extension at 72°C for 5 min. For amplification of ITS rDNA region, PCR conditions were: 95°C for 5 min; 25 cycles of denaturation at 95°C for 30 s; hybridization at 55°C for 1 min; extension at 72°C for 1 min; and a final extension at 72°C for 10 min. The amplicons were sequenced using paired-end sequencing on an Illumina MiSeq instrument at the Institute of Biotechnology at Cornell University in Ithaca New York, United States.

Raw sequence reads were de-multiplexed, low quality read ends were trimmed using Trimmomatic [41], and low-quality sequences were removed. QIIME2 (https://qiime2.org/) was used to perform the downstream diversity and taxonomy composition analysis. Corresponding paired end reads were merged and un-joined reads were discarded. Another quality processing was performed using ‘Deblur’ plugin in QIIME2 to remove chimeric sequences. Read quality profiles were visualized to retain high-quality sequences for 16S and ITS datasets. The remaining sequences were used to determine differences in bacterial and fungal communities between asymptomatic and symptomatic samples from root, shoot, soil, and rhizosphere, and to calculate the Shannon diversity index to obtain alpha and beta diversity statistics. Sequences were grouped to obtain operational taxonomic units (OTUs) with 97% similarity. The resulting OTUs were compared against the trained full-length Greengenes 13_8 OTUs database (http://greengenes.secondgenome.com/) for bacterial taxonomic classification. For ITS taxonomy analysis, a database was trained using the fungus sequences in UNITE (Fungal ITS) (https://unite.ut.ee/). Fungus OTUs were compared against the resulting database for taxonomic analysis of ITS sequences. A differential abundance analysis of the OTU was independently performed for bacterial and fungal datasets using a Kruskal-Wallis test with multiple correlation testing (FDR). The test was performed by separately comparing the asymptomatic and symptomatic samples within root, shoot, soil, and rhizosphere. The sample-specific differentially abundant OTUs were determined using a threshold *p*<0.01. The output files were visualized in QIIME2.

### Statistical analysis

The means and standard deviations of various soil elements from symptomatic and asymptomatic trees were analyzed using student’s T-test assuming unequal variances. A *p*-value of 0.05 was used as a threshold to declare significant differences. Similarly, the monthly means from different weather variables were compared against five-year averages using a student’s T-test, assuming unequal variances with significant *p*-value threshold of less than 0.05. The differential abundance of bacterial and fungal classes were analyzed using the Kruskal-Wallis test with multiple correlation testing (FDR). Class enrichment data was retrieved from QIIME2 output and a principal component analysis (PCA) was performed in R (https://www.r-project.org/) to visualize sample-specific variation. The ggplot2 package in R was used to plot PAC biplots.

## Results

### Physical and chemical composition of soil

**Fig 2:**
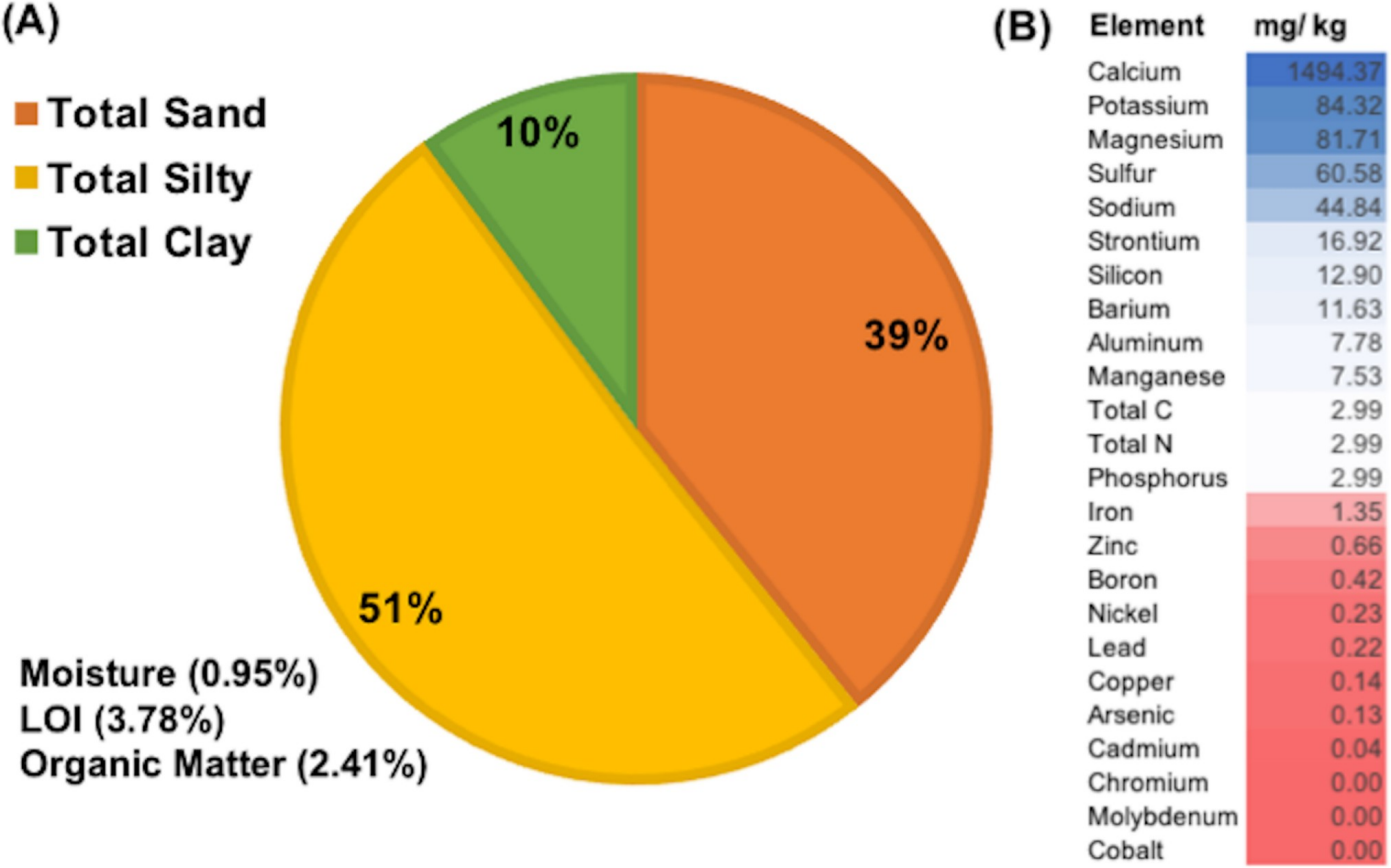
Composition of the bulk soil collected across the experimental apple orchard in a ‘HoneyCrisp’ block with rapid apple decline. (A) Physical properties and (B) Chemical composition of soil samples ranging from the highest (blue) to the lowest (red) order.

Analysis of the physicochemical properties of bulk soil showed that the experimental orchard area had silty loam soil (Fig 2A) with large proportions of sand and silt fractions (39.3% and 50.75% respectively), a pH of 6.8, total N of 0.14%, total C of 1.5%, and 2.4% organic matter. The quantities of various macro- and micronutrients showed a wide range in different soil elements (Fig 2B). In general, both macro- and micronutrients in soil from asymptomatic and symptomatic trees were homogeneous (Table 1). However, calcium (1839.6 mg/Kg) and manganese (8.5 mg/Kg) were present at higher concentrations in soil of symptomatic trees, and sulfur (82.4 mg/Kg) was slightly higher in soil of asymptomatic trees (Table 1). Organic matter, total nitrogen, and elements such as phosphorus, potassium, magnesium, and boron were at low concentrations in the experimental orchard area.

**Table 1:** Physicochemical properties and nutrient profiles from 0–20 cm topsoil in a commercial apple orchard in Wayne County, NY from RAD asymptomatic and symptomatic apple trees.

	**Moisture**	**pH**	**Aluminum**	**Calcium**	**Copper**	**Iron**	**Potassium**
	**(%)**		(mg/Kg)				
**Asymptomatic**	0.72 ± 0.11	6.8 ± 0.04	10.0 ± 5.9	1839.6 ± 273.6	0.43 ± 0.07	1.57 ± 0.6	90.0 ± 0.13
**Symptomatic**	0.69 ± 0.1	6.85 ± 0.4	10.48 ± 7.1	1713.39 ± 252.5	0.42 ± 0.05	1.63 ± 0.8	83.39 ± 19.3
**T test**	0.4089	0.2189	0.0944	0.7081	0.083	0.1293	0.7207
**P value (P < 0,05)**	0.6948	0.833	0.9274	0.5018	0.9361	0.9007	0.4945
	**Magnesium**	**Manganese**	**Molybdenum**	**Sodium**	**Phosphorus**	**Sulfur**	**Zinc**
	(mg/Kg)						
**Asymptomatic**	102.2 ± 29.3	8.5 ± 1.24	0.15 ± 0.01	43.4 ± 6.3	3.5 ± 0.3	80.3 ± 31.7	0.9 ± 0.06
**Symptomatic**	105.19 ± 41.7	7.13 ± 1.2	0.14 ± 0.01	43.17 ± 10.9	3.17 ± 0.7	82.39 ± 47.6	0.84 ± 0.1
**T test**	0.1162	1.81	1	0.0443	0.7685	0.0656	0.7943
**P value (P < 0.05)**	0.9108	0.1133	0.3506	0.9659	0.4673	0.9495	0.4531

### Weather data in the vicinity of the experimental orchard

**Fig 3:**
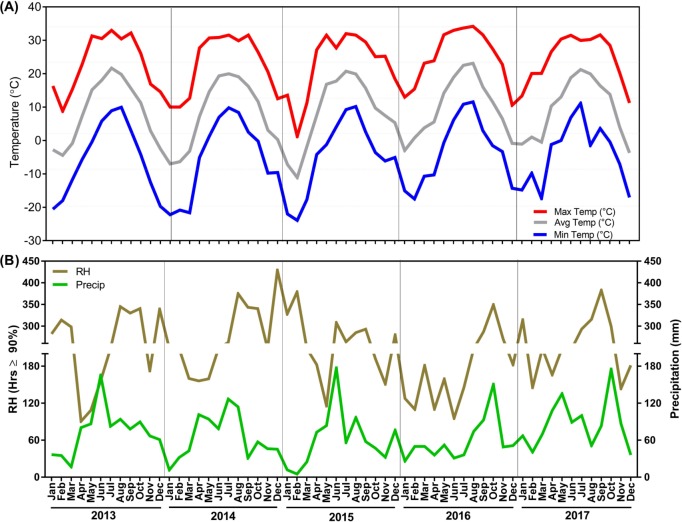
Maximum, average, and minimum, temperatures (A), precipitation and relative humidity (B) obtained from the weather stations located at Phelps, Farmington, and Sodus, New York from 2013 to 2017.

Temperatures (maximum, average, and minimum), precipitation, and relative humidity values were collected for three weather stations (Phelps, Farmington-, and Sodus, New York) from 2013 to 2017 (Fig 3; S1 Table). Temperatures showed similar trends in 2013 and 2014. Minimum and maximum temperatures were -20.6°C and 26.1°C in 2013 and -22.2°C and 27.7°C in 2014 during the winter months (October to April). Summer (May to September) maximum and minimum temperature were 33°C and -0.4°C in 2013 and 31.6°C and 1.3°C in 2014, respectively. Abnormally cold temperatures were observed in December 2014 to March 2015. A record minimum temperature of -23.9°C was noted in February 2015. Also, the number of extremely cold days with temperatures below -10°C was comparatively much higher in winter of 2014 and 2015 (S2 Table). In contrast, abnormal warming was registered throughout 2016, represented by an unusually warm winter and a hot summer (S1 Table). Temperatures ranged from a minimum of -15.1°C and an average of -3°C in January to a maximum of 34.2°C and and average of 23.1°C in August of 2016. Slightly warmer weather was also noticed in 2017.

Analysis of weather data for total rainfall indicated alternate rainfall patterns over the five year period. Precipitation peaks (above 120 mm) were generally observed from May to August (S2 Fig). Summers of 2013 and 2015 had relatively higher rainfall than other years. Highest rainfall was documented in June 2015 (177.1 mm) followed by 174.9 mm in October 2017. Average rainfall decreased in 2014 and the rainfall season shifted from June to October in 2016 and 2017, which resulted in a drier pre-harvest season (S2 Fig). Severe drought was also reported in 2016. Relative humidity correlated with precipitation data.

### Internal necrotic symptoms across the graft union

**Fig 4:**
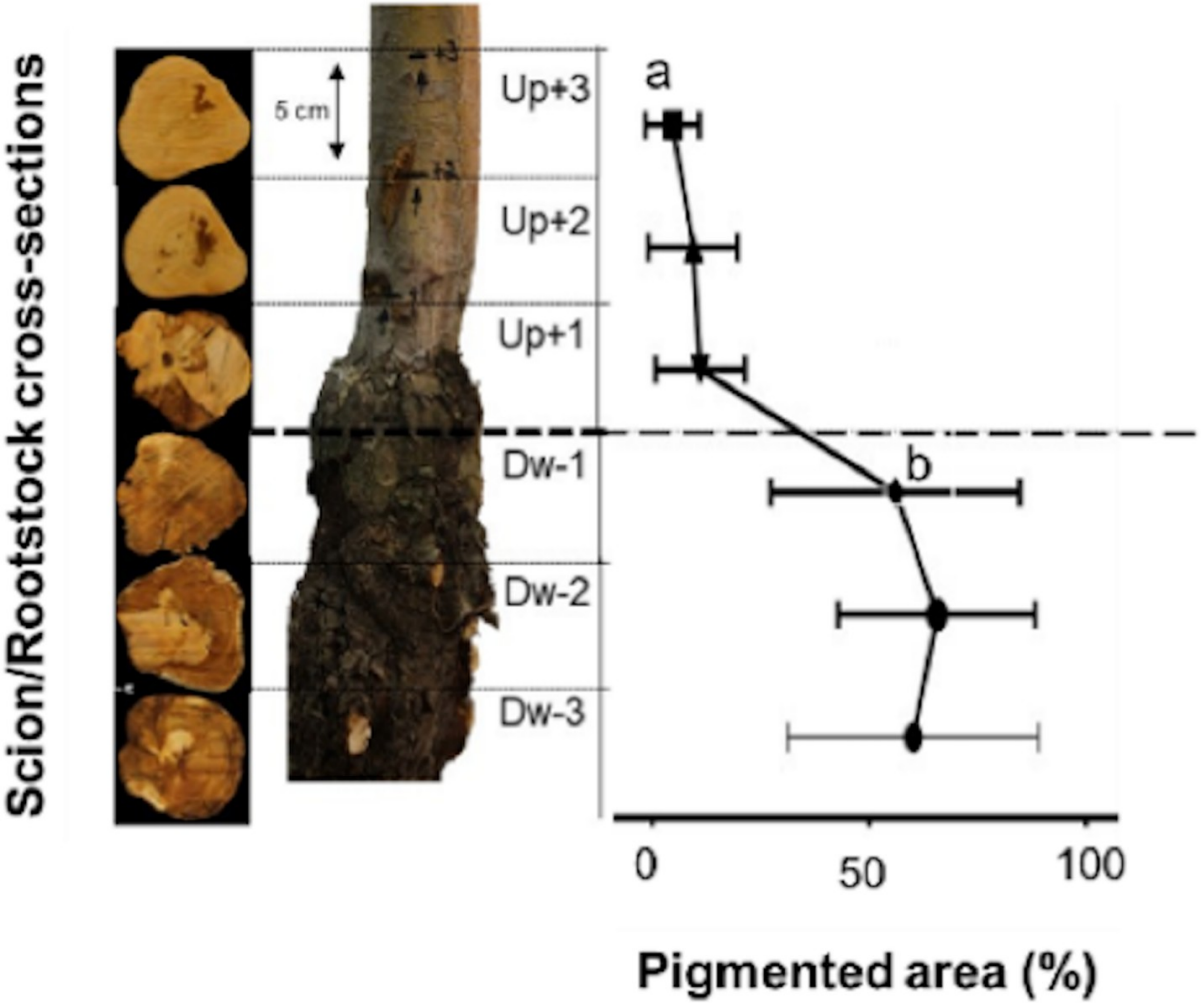
Scion and rootstock cross sections from ‘HoneyCrisp’ apple trees grafted onto M.9 (clone NIC 29) with rapid apple decline. Three cuts of 5 cm were made above and below the graft union at the scion-rootstock junction. The following discoloration scale was used to estimate healthy and pigmented necrotic wood area in symptomatic apple trees: DS = 1, external wood discoloration, DS = 2, internal wood discoloration, DS = 3, co-occurrence of external and internal wood discoloration, DS = 4, edge-shaped discoloration (<50%), DS = 5, edge-shaped discoloration (>50%), and DS = 6, circular discoloration.

The presence of abnormal wood and potential signs of pathogen and insect damage were examined at three different positions above and below the graft union. Dark brown wood discoloration was observed in all cross-sections, but the proportion and sites of internal decay/damage varied across the three sampling points (Fig 4). Wood decay was predominantly present below the graft union (*p* < 0.01) (Fig 4). The main decay symptoms of dark brown wood discoloration represented more than 50% of the total area in cross-sections DW-1, DW-2, and DW-3. Visual inspection of the necrotic lesions also indicated that symptoms initiated in the bark or near the vascular cambium of the rootstock and tended to move towards the heartwood. In contrast, no or minimal internal decay was observed in the cross-sections above the graft union (UP+1, UP+2, and UP+3) (Fig 4), suggesting that damage started at the graft union and moved inwards and then upwards in the declining trees.

**Fig 5:**
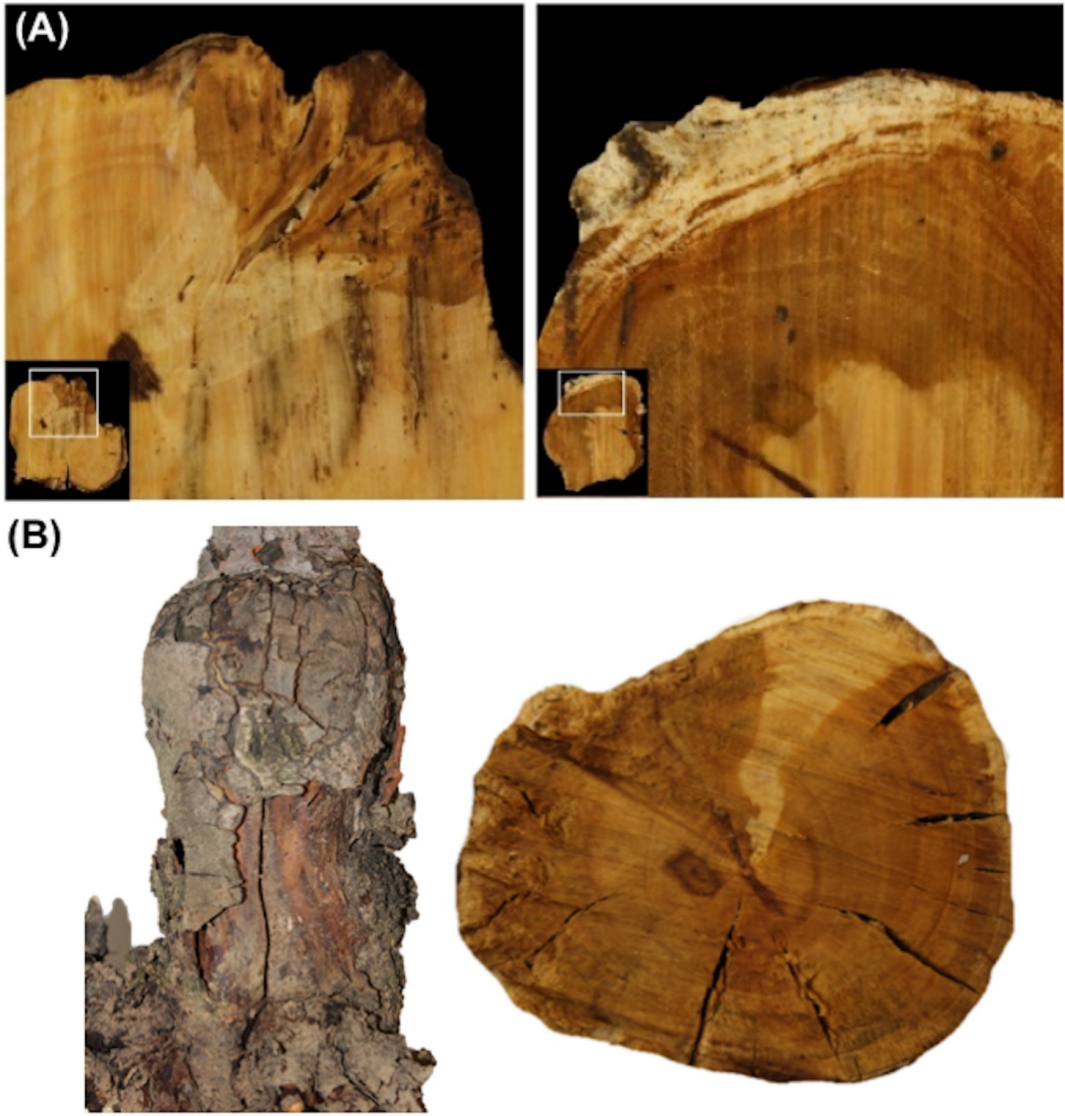
Visual symptoms of wood decay in declined apple trees. (A) Wood cross-sections showing rot-like decay around the graft union, (B) Cracks in the bark and internal wood from declined apple trees.

Visual observations showed the presence of spongy and white rot-like decay in the cross-sections (Fig 5A). Spongy decay was present in 82% of cross-sections below the graft union, and in 8% of the tree trunk; whereas white rot-like decay was present in 77% of the cross-sections below the graft union and exclusively in UP+1 (4% of the tree trunk samples). Co-occurrence of spongy and white rot-like decay were observed in 44% of cross-sections below the graft union, and 61% of the rootstock samples tested displayed cracks in the bark and in the internal wood (Fig 5B). Wood boring insects were not detected in the rootstock or scion wood.

### Detection of viruses in symptomatic and asymptomatic apple trees

Out of the 58 shoot and root samples collected from symptomatic and asymptomatic trees, 11 were positive for apple stem pitting virus (*ASPV*) and/or apple chlorotic leafspot virus (*ACLSV*) in DAS-ELISA test (Table 2). Shoots of asymptomatic trees were also found to be infected with *ASGV* while none of the shoots of symptomatic trees carried any of the viruses tested. DAS-ELISA also showed the presence of *ASPV* and *ACLSV* in two root samples of asymptomatic trees, and the co-occurrence of *ASGV* and *ACLSV* in two root samples of symptomatic trees.

**Table 2:** Detection of different viruses in root and shoot tissue of asymptomatic and symptomatic apple trees.

			Number of Positive samples					
Tissue	Status	Total Samples	ASGV	ASPV	ACLSV	ToRSV	APMV	TRSV
Shoots	Asymptomatic	16	-	5	-	-	-	-
	Symptomatic	13	-	-	-	-	-	-
Roots	Asymptomatic	16		2	2	-	-	-
	Symptomatic	13	-	2	2	-	-	-

Here, *ASGV* = Apple stem grooving virus, *ASPV* = Apple stem pitting virus, *ACLSV* = Apple chlorotic leafspot virus, *ToRSV* = Tomato ringspot virus, *APMV* = Apple mosaic virus, and *TRSV* = Tobacco ringspot virus.

### Bacterial and fungal classes in symptomatic and asymptomatic apple trees

**Fig 6:**
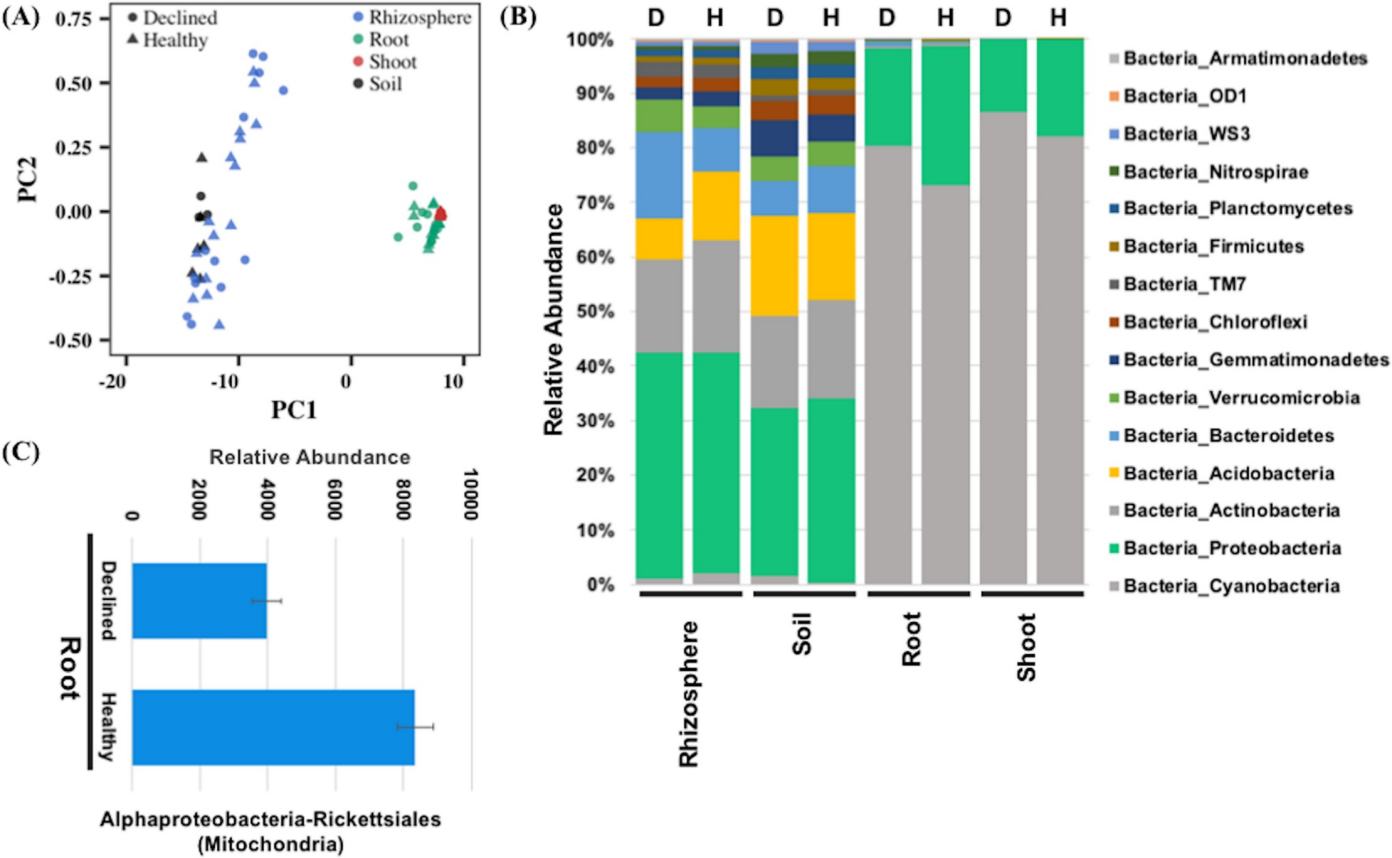
Analysis of bacterial communities in root, shoot, soil, and rhizosphere samples from asymptomatic and symptomatic ‘HoneyCrisp’ apple trees as identified by sequencing 16S regions. (A) Biplot from principal component analysis of the diversity indices obtained from 16S analysis of different samples, (B) Abundance of different bacterial classes in the rhizosphere, soil, root, and shoot samples, (C) Bacterial classes that show a significantly differential abundance between healthy and declined apple trees.

High-throughput sequencing was used to identify the bacterial and fungal communities in symptomatic and asymptomatic apple trees. A total of 640 bacterial OTUs were detected from the 16S sequencing of shoot, root, soil, and rhizosphere samples (S1 File). The maximum number of OTUs was identified in rhizosphere (n = 637) followed by soil (n = 506) and root (n = 367) samples. Shoot samples had the minimum, 17 OTUs. Only 16 OTUs (2.5%) were shared between all samples (S3 Fig). However, the percentage of shared OTUs between soil and rhizosphere samples and between shoot and root samples were comparatively much higher. All shoot OTUs were present in the root samples and 78.9% of OTUs from the soil and rhizosphere samples were identical (S3 Fig). The distinction between plant tissues and soil-rhizosphere was also apparent from the Shannon diversity index of the bacterial communities in these samples. Shoot and root samples had low Shannon diversity indices of 2.31 and 2.75, respectively. In contrast, rhizosphere and soil samples had comparatively higher diversity, with Shannon index values of 8.94 and 9.35, respectively. This was also evident from the multivariate analysis of diversity fractions from shoot, root, soil, and rhizosphere. There was a clear separation between the plant samples from soil-rhizosphere samples (Fig 6A), indicating that the bacterial communities in the plant tissues were considerably different from the soil and rhizosphere.

The 640 OTUs represented 35 broad classes of bacteria (Fig 6B; S1 File). Cyanobacteria and proteobacteria were prominent bacterial classes in the shoot and root samples and together constituted about 99.8% and 98% of the total identified OTUs (S1 File). The root samples, in addition to the shoot-abundant classes, showed high enrichment of bacteroidetes and firmicutes categories. Relatively different sets of bacterial classes were abundant in soil and rhizosphere samples. Proteobacteria, acidobacteria, and actinobacteria were dominant in soil samples, whereas bacteroidetes, cloroflexi, gemmatimonadetes, and verrucomicrobia classes, in addition to soil-abundant bacterial classes, were enriched in the rhizosphere (S1 File). Proteobacteria constituted about 31.9% of soil bacteria, whereas both acidobacteria and actinobacteria constituted about 17% of the total bacterial fractions in soil. Proteobacteria were also comparatively higher (40.6%) than acidobacteria (9.8%) and actinobacteria (18.6%) in the rhizosphere.

To discern the role of bacteria in RAD, we analyzed the differential abundance of identified bacterial OTUs in symptomatic and asymptomatic samples from the shoot, root, soil, and rhizosphere using a Kruskal-Wallis test. The symptomatic and asymptomatic samples were compared separately to determine the significantly abundant classes among them. No significant difference in abundance of bacterial classes was observed between symptomatic and asymptomatic samples from shoot, soil, and rhizosphere. Only root samples showed differential abundance for two OTUs related to alphaproteobacteria, which belongs to rickettsiales at a higher taxonomic level (Fig 6C).

**Fig 7:**
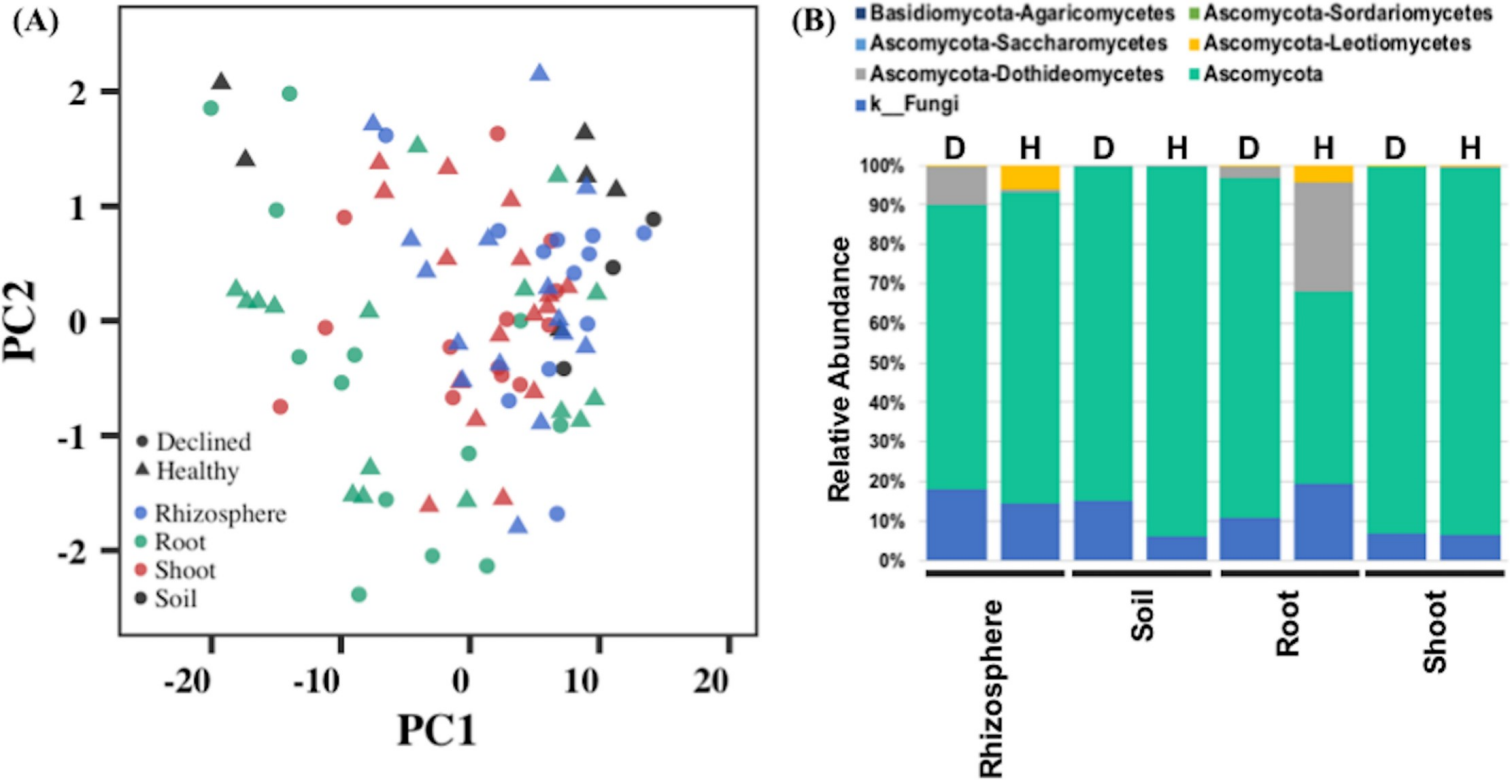
Analysis of fungal communities in root, shoot, soil, and rhizosphere samples from asymptomatic and symptomatic ‘HoneyCrisp’ apple trees as identified by sequencing ITS regions. (A) Biplot from principal component analysis of the diversity indices obtained from ITS sequence analysis of various samples, (B) Abundance of different fungal classes in the rhizosphere, soil, root, and shoot samples.

Fungal ITS sequences detected many fungal classes in the shoot, root, soil, and rhizosphere samples (S2 File). Rhizosphere samples had the most fungal classes, followed by root samples. Shoot and soil had comparatively less variation in fungi. An analysis of diversity index indicated no clear distinction between samples from the two plant tissues, soil, and rhizosphere (Fig 7A). A total of 18 fungal classes were identified, mainly representing the Basidiomycota and Ascomycota fungal populations in these samples (Fig 7B). Soil and shoot samples showed the presence of only Ascomycota, whereas root and rhizosphere had both Ascomycota and Basidiomycota fungus groups. The Ascomycota sub-classes dothideomycetes, leotiomycetes, and sordariomycetes were present in all samples except shoot, which did not show presence of sordariomycetes sub-class (S2 File). In addition, Ascomycota sub-class saccharomycetes was detected only in root and rhizosphere samples. Basiodiomycota had a single sub-class, agaricomycetes, in both root and rhizosphere samples. We also compared the fungal class enrichment in symptomatic and asymptomatic apple trees. However, differential abundance analysis showed no significant enrichment for any fungal class between the symptomatic and asymptomatic samples from shoot, root, soil, and rhizosphere.

## Discussion

Tree decline/death is often caused by diseases such as rootstock fire blight, phytophthora root and crown rots, or apple replant disease, or abiotic factors like soil wetness, extreme cold, spring and fall freezes, or drought, as well as injuries to trunk, graft union, crown and roots by rodents or insect borers. However, rapid or sudden decline of established apple trees (RAD), a recent concern to the apple industry, is diagnosed when symptoms do not match any of the above. We addressed this concern by conducting a comprehensive study to identify the role of soil nutrition, weather conditions, fungal and bacterial communities, as well as viruses in rapid decline of established ‘HoneyCrisp’ trees grafted onto the rootstock M.9 (clone NIC 29). Analysis of weather variables indicated abnormal trends at the experimental orchard site over the five-year growth period with December 2014, January, February and March 2015 being exceptionally cold. According to National Climate Report from the National Oceanic and Atmosphoric Administration (NOAA) National Centers for Environmental Information of the US, February 2015 was the third-coldest February on record in the region since 1934. In 2015, there were heavy rains between June-July followed by a severe drought in 2016 during the apple growing season. A severe temperature drop followed by a moderately warmer winter may have caused direct damage to apple trees or indirectly made them more susceptible to biotic and abiotic stresses [8, 9]. Both significant loss of trees and decrease in yield have previously occurred due to freezing temperatures in the United States and Canada [7, 42–45]. Cold injury symptoms become apparent in the spring following a hard winter with trees exhibiting stunted growth, wilting, and death. Rainfall data also indicated one drought instance in the area in 2016. Although no study has linked RAD with drought, general water stress can cause severe damage by reducing tree growth, root damage, and senescence [12–16, 46]. The occurrence of severe cold followed by drought, or either individually, might not directly cause RAD, but could have weakened the trees and led to the proliferation of insects and infection by opportunistic pathogens. Although the general soil fertility status was not optimal for commercial apple production [47], differences between soil nutrition profiles from symptomatic and asymptomatic apple trees were not significant, ruling out any potential role of nutritional stress in RAD in the experimental orchard. The concentrations of organic matter, total nitrogen, phosphorus, potassium, magnesium, and boron were all low in the experimental orchard area. Meanwhile, sulfur concentration was nearly two-fold over the recommended amount [47]. Nutritional differences might not directly contribute, but can exacerbate the impact of other stress factors such as weather extremes, insects, and pathogens, therefore increasing the chances of decline of apple trees. In some rootstock and scion combinations, a weak graft union can be impacted under extreme abiotic stresses, leading to a slow collapse of the tree. Also, rootstocks with shallow root-systems in high density plantings may have limited access to nutrients and water and thus be unable to support the heavy crop, foliage, and biomass under extreme weather, leading to decline and death of trees. The negative effect can be exacerbated in soils with poor water holding capacity.

The presence of necrotic lesions and wood decay were among the potential signs of declining apple trees in the orchard. These symptoms followed a specific dispersion pattern from bark or vascular cambium towards the heartwood. Also, the wood decay and discoloration mainly occurred below the graft union and progressed downwards. These observations indicated that the rootstock seems to be the originating point of the decline. Previous reports have implicated incompatibility between rootstock and scion as a potential trigger for RAD [2]. Poor vascular connections, phloem degeneration, and vascular discontinuity [48] can cause such graft incompatibility. However, the pattern of wood rot and discoloration in the trees examined in this study appears to be different from that seen with scion-rootstock incompatibility, which is generally equally dispersed above and below the graft union. The exact cause of necrotic lesions and discoloration only in rootstock wood is yet unclear, but it could involve multiple pathogens in RAD. Some microbes can also cause root rots and internal clotting of vascular tissues that lead to tree decline and ultimately death [22, 49, 50]. Rootstock fire blight caused by *Erwinia amylovora* and root or crown rot caused by *Phytophthora* and *Pythium* species can lead to tree death by infecting the root system. However, we did not identify symptoms or pathogens of common soil-borne root diseases or apple rootstock blight in any of the declining ‘HoneyCrisp’ trees.

Apple pathogenic viruses can potentially result in decline of trees with similar symptoms as observed for RAD. Tree decline from latent viruses, usually transmitted by grafting and top working, is an emerging commercial problem [4]. There were no differences between healthy-looking and declined plants for the presence of the stem pitting, stem grooving, apple chlorotic leaf spot, or apple mosaic, the most common latent viruses of apples. Tomato ringspot virus, which can be involved in apple tree decline and death, was not found in any of the declining ‘HoneyCrisp’ trees. Similarly, tobacco ringspot virus was not identified in this study. Fungal pathogens cause several soil-borne diseases in tree crops, resulting in symptoms similar to those observed in declining apple trees. For example, a vascular fungus, *Ceratocystis fimbriate* can kill mango trees two months after the initial infection, causing mango sudden decline or mango wilt [51]. In our study, several classes of bacterial and fungal communities were present in root, shoot, soil, and rhizosphere samples from apple trees. Similar bacterial and fungal classes have been detected previously in different apple tissues and soil types [38, 52–53]. For instance, both Basidiomycota and Ascomycota fungus classes were detected in microbiomes of the shoots and soil samples in apple trees [38, 53]. Similarly, bacterial classes related to proteobacteria, actinobacteria, and acidobacteria were also detected in these studies. These probably represent the most common bacterial and fungal classes in apple orchards from New York soils. However, future studies can more clearly define the enrichment of these microbiome populations in the apple cultivation area. Differential abundance analysis of ITS sequences of shoot, root, soil, and rhizosphere samples showed no significant enrichment of any fungal species in RAD affected apple trees. However, 16S sequencing identified a single bacterial class, ‘alphaproteobacteria-rickettsiales’, with differential abundance in the roots of healthy-looking and declined apple trees. These bacteria types were less abundant in the declined samples. The relative abundance of proteobacteria usually declines in water-limiting soils [54–56], suggesting a difference in the moisture level in roots of healthy-looking and declined trees. However, it is difficult to determine at this point whether roots from declining apple trees endured low moisture at the time of sampling or low moisture resulted from the severe drought in 2016. Nonetheless, these observations indicate the role of water-limiting conditions towards the rapid decline of apple trees. This can provide a focus for future RAD research to further explore the roles of soil and plant water status, taking into consideration the drought tolerance of different rootstocks and their susceptibility to rapid decline. Moreover, differences in root system architecture of the rootstocks can also be studied under extreme water stress conditions in different soil types for its role in RAD. For instance, shallow rooted rootstocks can be at a disadvantage in non-irrigated orchards with a low water table, whereas rootstocks with deeper and more vigorous roots may be more tolerant to drought stress conditions. Parasitic nematodes such as root lesion, root knot, and dagger nematodes can also infect the root system of apple trees, resulting in leaf chlorosis, stunted growth of trees and poor yields. However, we did not evaluate the presence of pathogenic nematodes in RAD affected orchards. RAD symptoms are distinct from those of apple replant disease. A complex of multiple species of fungi and oomycetes including *Rhizoctonia*, *Phytophthora*, and *Pythium* species, and root lesion nematodes causes apple replant disease [31], characterized by reduced productivity in orchards repeatedly planted with the same or closely related fruit trees. Symptoms of replant disease, including uneven or stunted growth of trees with short internodes, are visible shortly after planting new trees. When a tree is uprooted, discolored roots, root tip necrosis, and reduced root biomass can be seen. Young trees may die within the first year. Many will survive but overall fruit production and quality are reduced [57].

In conclusion, we did not find any statistically significant differences in soil and weather profiles of healthy-looking and declined trees. Similarly, no particular fungi and viruses were associated with the symptomatic trees. A single class of proteobacteria showed differential abundance between symptomatic and asymptomatic samples, suggesting a possible role of water-limiting conditions. Similarly, the role of different opportunistic or previously unknown pathogens should not be excluded. We speculate that the onset of RAD symptoms is much later than the actual cause of the decline. The findings in this study, however, will require further validation in different declining orchards that have diverse scion-rootstock combinations and different soil types and weather conditions.

## Supporting information

S1 Fig The ‘HoneyCrisp’ orchard block with rapid apple decline (RAD) selected for this study in Wayne County, NY. The arrows identify row numbers in the orchard block (A). Schematic distribution of asymptomatic (green) and RAD symptomatic (yellow) apple ‘HoneyCrisp’ trees in the study orchard (B-C). Samples from three sets of trees (two asymptomatic neighboring one symptomatic) were collected in row R2, and 10 asymptomatic and 10 symptomatic trees were randomly selected in row R3.
(TIFF)

S2 Fig The five-year average rainfall data for the ‘HoneyCrisp’ orchard block with rapid apple decline (RAD). Twelve month precipitation (mm) data was obtained for Phelps, Farmington, and Sodus, New York from 2013 to 2017.
(TIFF)

S3 Fig Venn-diagram showing the percentage of shared and unique bacterial communities. The analysis was conducted in rhizosphere, soil, root, and shoot samples from asymptomatic and symptomatic samples. Different colors represent the number of unique and shared bacterial communities between root, shoot, rhizosphere, and soil.
(TIFF)

S1 Table Weather dataset obtained from 2013 to 2017 from Network for Environment and Weather Applications (NEWA). Data represent the mean values of monthly observations obtained from the weather stations located in Phelps, Farmington, and Sodus, New York.
(DOCX)

S2 Table Temperatures over the four winter months (December-March) over five years at three locations near the studied orchards. The average temperature was obtained for all four months. The number of extreme cold days were calculated by counting number of days with temperatures below -10°C.
(DOCX)

S1 File Different bacterial classes detected by 16S sequencing. Analysis was conducted using rhizosphere, soil, root, and shoot samples from asymptomatic and symptomatic ‘HoneyCrips’ trees.
(XLSM)

S2 File Different fungus classes detected by ITS sequencing. Analysis was conducted using rhizosphere, soil, root, and shoot samples from asymptomatic and symptomatic ‘HoneyCrisp’ trees.
(XLSM)
