# Supplementary figures and images for: Potential role of weather, soil and plant microbial communities in rapid decline of apple trees
Source: PLoS One. 2019 Mar 6;14(3):e0213293. doi: 10.1371/journal.pone.0213293 (PMC6402675; doi:10.1371/journal.pone.0213293)

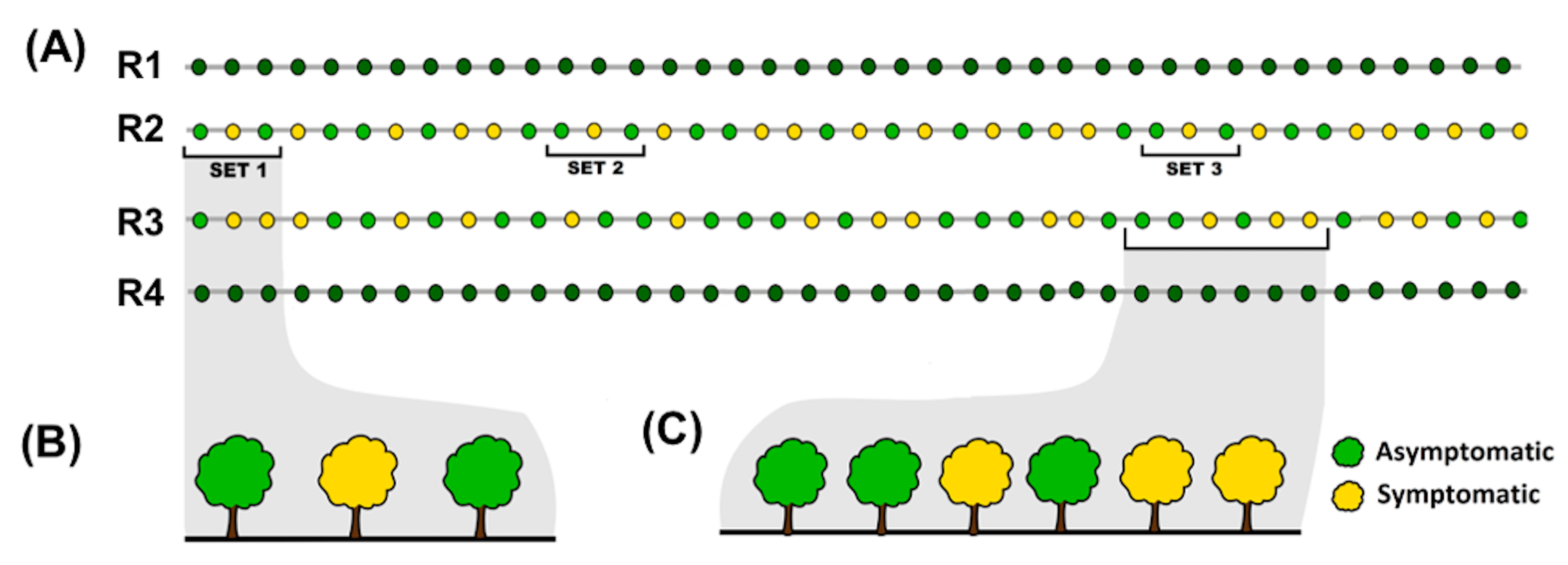

Supplement: S1 Fig — The arrows identify row numbers in the orchard block (A). Schematic distribution of asymptomatic (green) and RAD symptomatic (yellow) apple ‘HoneyCrisp’ trees in the study orchard (B-C). Samples from three sets of trees (two asymptomatic neighboring one symptomatic) were collected in row R2, and 10 asymptomatic and 10 symptomatic trees were randomly selected in row R3. (TIFF) [file pone.0213293.s001.tiff]

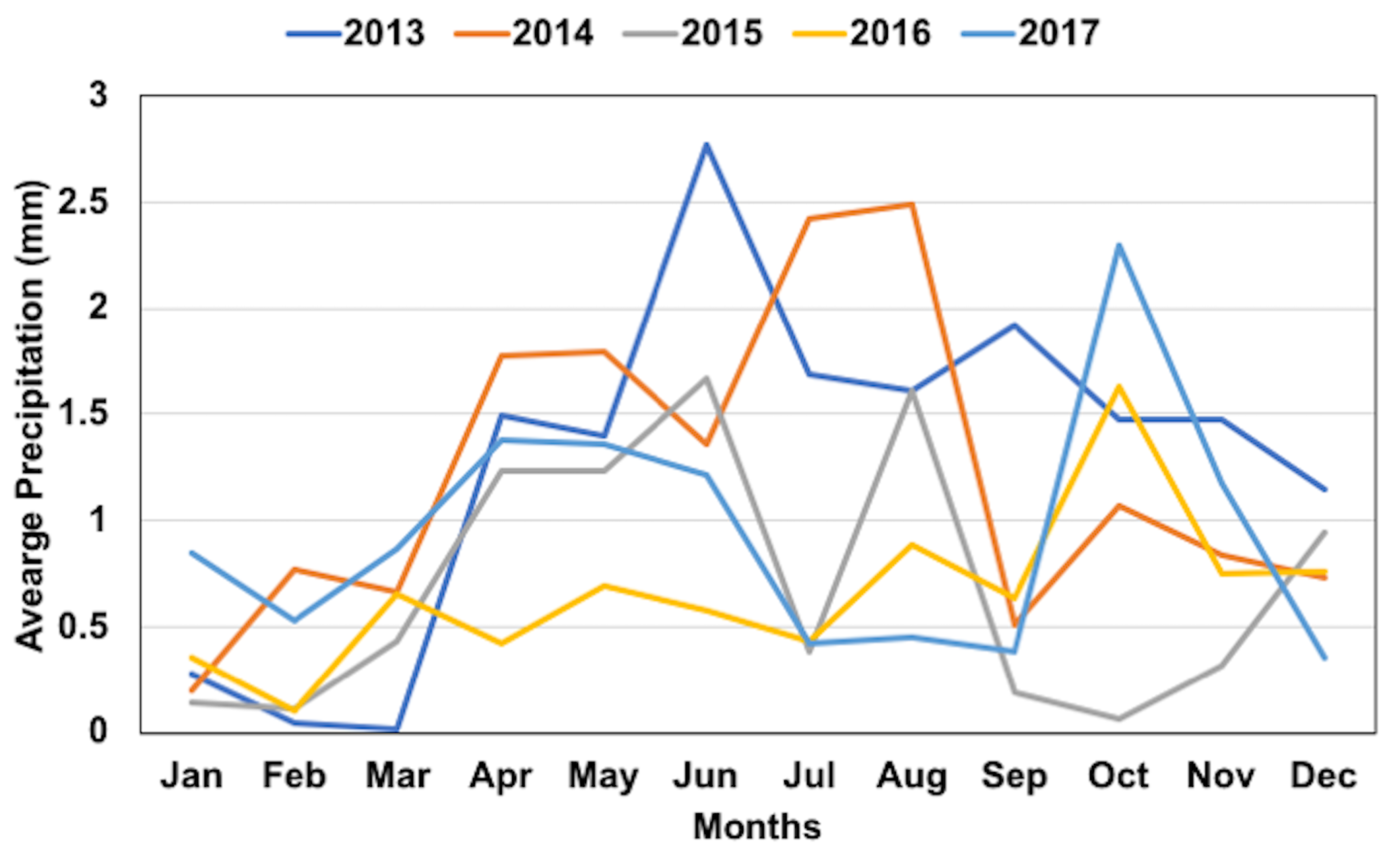

Supplement: S2 Fig — Twelve month precipitation (mm) data was obtained for Phelps, Farmington, and Sodus, New York from 2013 to 2017. (TIFF) [file pone.0213293.s002.tiff]

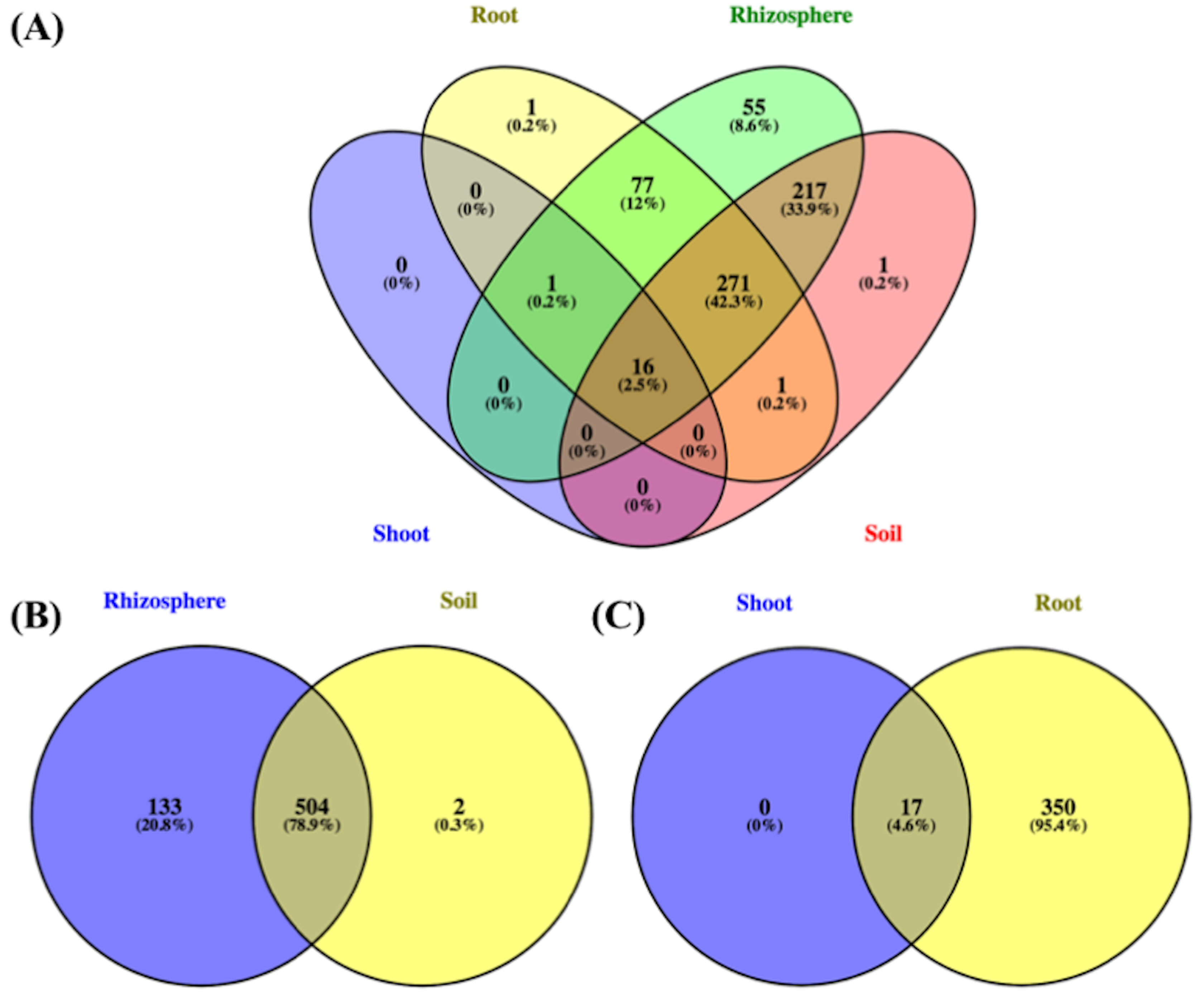

Supplement: S3 Fig — The analysis was conducted in rhizosphere, soil, root, and shoot samples from asymptomatic and symptomatic samples. Different colors represent the number of unique and shared bacterial communities between root, shoot, rhizosphere, and soil. (TIFF) [file pone.0213293.s003.tiff]
