# Supplementary material for: Potential role of weather, soil and plant microbial communities in rapid decline of apple trees
Source: PLoS One. 2019 Mar 6;14(3):e0213293. doi: 10.1371/journal.pone.0213293 (PMC6402675; doi:10.1371/journal.pone.0213293)
Supplement: S1 Table — Data represent the mean values of monthly observations obtained from the weather stations located in Phelps, Farmington, and Sodus, New York. (DOCX) [file pone.0213293.s004.docx]

**S1 Table.** Compilation of NEWA weather dataset obtained from 2013 to 2017. Data represent the mean value of monthly observations obtained from the weather stations located in Phelps - NY, Farmington- NY, and Sodus- NY.

| **Year** |  | **January** | **February** | **March** | **April** | **May** | **June** | **July** | **August** | **September** | **October** | **November** | **December** |
| --- | --- | --- | --- | --- | --- | --- | --- | --- | --- | --- | --- | --- | --- |
| **2013** | Max T (°C) | 16.3 ± 0.0 | 8.0 ± 0.0 | 15.4 ± 0.0 | 22.9 ± 0.2 | 31.3 ± 0.9 | 30.0 ± 1.0 | 33.0 ± 0.7 | 30.4 ± 0.9 | 32.2 ± 0.8 | 26.1 ± 0.5 | 16.9 ± 0.3 | 14.6 ± 0.5 |
|  | Avg T (°C) | - 2.7 ± 0.0 | - 4.4 ± 0.0 | - 0.8 ± 0.0 | 7.4 ± 0.4 | 15.2 ± 0.0 | 18.1 ± 0.4 | 21.7 ± 0.5 | 19.8 ± 0.4 | 15.6 ± 0.6 | 11.3 ± 0.5 | 2.9 ± 0.9 | - 2.5 ± 0.4 |
|  | Min T (°C) | - 20.6 ± 0.0 | - 18.0 ± 0.0 | - 11.7 ± 0.0 | - 5.6 ± 2.6 | - 0.4 ± 1.8 | 5.8 ± 1.6 | 8.9 ± 2.4 | 10.0 ± 1.5 | 3.0 ± 2.7 | - 4.2 ± 3.9 | - 12.6 ± 2.7 | - 19.7 ± 2.5 |
|  | Prec (mm) | 36.6 ± 0.0 | 35.0 ± 0.0 | 16.8 ± 0.0 | 80.5 ± 8.6 | 86.4 ± 8.6 | 165.2 ± 8.4 | 82.4 ± 22.8 | 93.8 ± 2.3 | 77.9 ± 50.3 | 89.5 ± 10.6 | 66.9 ± 28.2 | 60.9 ± 7.9 |
|  | RH (Hrs ≥ 90%) | 282.0 ± 0.0 | 314.0 ± 0.0 | 298.0 ± 0.0 | 90.5 ± 16.3 | 108.5 ± 89.8 | 156.5 ± 210 | 254.5 ± 136.5 | 345.0 ± 5.7 | 330.0 ± 28.3 | 340.5 ± 87.0 | 172.5 ± 26.2 | 339.0 ± 63.6 |
|  |  |  |  |  |  |  |  |  |  |  |  |  |  |
| **2014** | Max T (°C) | 10.0 ± 0.0 | 10.0 ± 0.7 | 127 ± 2.5 | 27.7 ± 0.6 | 30.7 ± 0.2 | 30.9 ± 0.2 | 31.6 ± 0.4 | 29.9 ± 0.6 | 31.6 ± 1.4 | 26.4 ± 0.7 | 20.6 ± 0.2 | 12.5 ± 0.0 |
|  | Avg T (°C) | - 7.0 ± 0.8 | - 6.3 ± 0.9 | - 3.2 ± 0.5 | 7.2 ± 0.2 | 14.6 ± 0.3 | 19.4 ± 0.2 | 20.0 ± 0.4 | 19.1 ± 0.3 | 16.2 ± 0.4 | 11.6 ± 0.4 | 3.1 ± 0.7 | 0.3 ± 0.8 |
|  | Min T (°C) | - 22.2 ± 3.6 | - 20.8 ± 2.7 | - 21.6 ± 4.0 | - 5.1 ± 1.3 | 1.3 ± 2.5 | 7.0 ± 1.0 | 9.8 ± 1.3 | 8.4 ± 1.6 | 2.5 ± 1.3 | - 0.2 ± 2.1 | - 9.8 ± 1.2 | - 9.6 ± 1.4 |
|  | Prec (mm) | 11.7 ± 0.7 | 32.0 ± 14.7 | 42.8 ± 5.2 | 101.3 ± 1.4 | 94.4 ± 19.6 | 78.6 ± 0.5 | 126.7 ± 27.3 | 113.9 ± 53.0 | 30.9 ± 2.9 | 57.3 ± 9.9 | 46.1 ± 3.4 | 45.5 ± 3.2 |
|  | RH (Hrs ≥ 90%) | 219.0 ± 15.6 | 223.5 ± 13.4 | 160.0 ± 5.7 | 156.0 ± 33.9 | 159.5 ± 55.9 | 239.5 ± 30.4 | 262.5 ± 21.9 | 375.0 ± 62.2 | 343.5 ± 54.4 | 340.5 ± 181.7 | 201.0 ± 42.4 | 429.0 ± 75.0 |
|  |  |  |  |  |  |  |  |  |  |  |  |  |  |
| **2015** | Max T (°C) | 13.6 ± 20.3 | 1.3 ± 0.4 | 11.3 ± 0.4 | 27.2 ± 0.1 | 31.5 ± 0.6 | 27.7 ± 0.3 | 32.0 ± 0.1 | 31.6 ± 0.6 | 29.5 ± 3.9 | 25.1 ± 0.4 | 25.2 ± 0.7 | 18.5 ± 0.2 |
|  | Avg T (°C) | - 7.2 ± 0.9 | - 11.1 ± 1.2 | - 1.8 ± 0.5 | 7.5 ± 0.0 | 16.8 ± 0.4 | 17.8 ± 0.3 | 20.7 ± 0.1 | 19.9 ± 0.3 | 15.7 ± 0.2 | 9.7 ± 0.2 | 7.5 ± 0.3 | 5.4 ± 0.4 |
|  | Min T (°C) | - 22.0 ± 2.7 | - 23.9 ± 2.2 | - 17.6 ± 2.5 | - 4.2 ± 0.2 | - 1.2 ± 1.4 | 4.1 ± 1.0 | 9.2 ± 1.2 | 10.2 ± 0.8 | 2.6 ± 4.4 | - 3.6 ± 1.0 | - 6.1 ± 1.8 | - 5.1 ± 0.4 |
|  | Prec (mm) | 11.9 ± 5.0 | 5.1 ± 1.4 | 24.9 ± 1.1 | 73.0 ± 3.1 | 83.6 ± 22.5 | 177.1 ± 37.4 | 56.3 ± 33.0 | 96.7 ± 41.5 | 57.7 ± 41.8 | 46.9 ± 41.8 | 32.3 ± 6.0 | 76.0 ± 9.1 |
|  | RH (Hrs ≥ 90%) | 328.0 ± 8.5 | 379.0 ± 5.7 | 238.5 ± 78.5 | 182.5 ± 65.8 | 115.7 ± 27.5 | 308.0 ± 7.2 | 263.7 ± 14.6 | 285.0 ± 15.6 | 293 ± 109.2 | 191.7 ± 37.5 | 151.0 ± 27.2 | 279.7 ± 7.5 |
|  |  |  |  |  |  |  |  |  |  |  |  |  |  |
| **2016** | Max T (°C) | 13.0 ± 1.2 | 15.4 ± 2.4 | 23.2 ± 0.6 | 23.9 ± 0.1 | 31.6 ± 1.0 | 33.0 ± 0.7 | 33.7 ± 0.5 | 34.2 ± 1.0 | 31.6 ± 3.1 | 27.5 ± 0.8 | 22.78 ± 1.6 | 10.6 ± 0.4 |
|  | Avg T (°C) | - 3.0 ± 0.5 | 0.8 ± 4.7 | 3.8 ± 0.3 | 5.6 ± 0.2 | 14.2 ± 0.1 | 18.9 ± 0.0 | 22.5 ± 0.3 | 23.1 ± 0.2 | 16.1 ± 0.5 | 11.6 ± 0.5 | 6.5 ± 0.4 | -0.9 ± 0.7 |
|  | Min T (°C) | - 15.1 ± 2.2 | - 17.5 ± 15.4 | - 10.7 ± 1.4 | - 10.3 ± 3.1 | - 0.7 ± 0.5 | 6.1 ± 1.3 | 10.9 ± 0.8 | 11.6 ± 1.3 | 3.0 ± 4.0 | - 1.5 ± 1.3 | - 3.3 ± 0.7 | - 14.3 ± 0.3 |
|  | Prec (mm) | 26.2 ± 6.3 | 49.9 ± 44.9 | 49.9 ± 7.9 | 35.9 ± 3.8 | 52.2 ± 9.0 | 31.9 ± 16.7 | 36.2 ± 11.3 | 74.2 ± 4.1 | 92.8 ± 71.7 | 150.5 ± 21.7 | 49.0 ± 14.3 | 51.3 ± 14.1 |
|  | RH (Hrs ≥ 90%) | 127.7 ± 88.7 | 110 ± 100.5 | 181.0 ± 36.4 | 110.3 ± 28.5 | 159.0 ± 25.2 | 95.7 ± 25.4 | 145.3 ± 30.7 | 225.3 ± 31.6 | 287 ± 60.2 | 349.3 ± 11.9 | 269.7 ± 49.6 | 182.3 ± 59.7 |
|  |  |  |  |  |  |  |  |  |  |  |  |  |  |
| **2017** | Max T (°C) | 13.4 ± 1.6 | 20.1 ± 0.3 | 20.1 ± 0.5 | 26.6 ± 0.4 | 30.4 ± 0.9 | 31.5 ± 0.9 | 29.9 ± 0.3 | 30.2 ± 0.6 | 31.6 ± 1.7 | 28.5 ± 1.4 | 20.2 ± 2.3 | 11.3 ± 0.1 |
|  | Avg T (°C) | - 1.1 ± 0.5 | 1.0 ± 0.4 | - 0.5 ± 0.5 | 10.3 ± 0.3 | 13.0 ± 0.2 | 18.8 ± 0.2 | 21.2 ± 0.8 | 19.9 ± 0.9 | 16.4 ± 0.5 | 13.8 ± 0.5 | 4.0 ± 0.5 | - 3.7 ± 0.8 |
|  | Min T (°C) | - 14.8 ± 2.5 | - 9.8 ± 1.1 | - 17.3 ± 2.7 | - 1.2 ± 1.5 | 0.0 ± 0.7 | 6.9 ± 1.1 | 11.1 ± 1.4 | - 1.4 ± 15.2 | 3.6 ± 4.1 | - 0.4 ± 1.5 | - 7.0 ± 1.0 | - 17.0 ± 1.7 |
|  | Prec (mm) | 67.1 ± 6.8 | 40.7 ± 5.3 | 70.0 ± 5.0 | 107.9 ± 7.3 | 135.2 ± 23.1 | 89.0 ± 12.7 | 99.8 ± 86.0 | 51.2 ± 26.7 | 83.2 ± 69.6 | 174.9 ± 22.3 | 86.9 ± 11.5 | 35.9 ± 14.1 |
|  | RH (Hrs ≥ 90%) | 314.3 ± 61.0 | 145.3 ± 33.5 | 230.0 ± 38.3 | 166.0 ± 32.6 | 248.3 ± 36.2 | 232.3 ± 41.0 | 293.3 ± 117.0 | 315.7 ± 121.8 | 382.7 ± 131.2 | 299.3 ± 194.7 | 143.7 ± 102.2 | 181.3 ± 169.4 |
