# Supplementary material for: Potential role of weather, soil and plant microbial communities in rapid decline of apple trees
Source: PLoS One. 2019 Mar 6;14(3):e0213293. doi: 10.1371/journal.pone.0213293 (PMC6402675; doi:10.1371/journal.pone.0213293)
Supplement: S2 Table — The average temperature was obtained for all four months. The number of extreme cold days were calculated by counting number of days with temperatures below -10°C. (DOCX) [file pone.0213293.s005.docx]

**S2 Table.** The temperature trend over the four winter months (December-March) over five years at three locations near the studied orchards. The average temperature was obtained for all four months. The number of extreme cold days were calculated by counting number of days having temperature below -10^0^C.

|  |  | **Average (December-March)** | **SE** | **Min** | **Max** | **No. of Days (less than -10 C)** |
| --- | --- | --- | --- | --- | --- | --- |
| Sodus | 2013 | -2.20 | 0.35 | -11.72 | 10.22 | 1 |
|  | 2014 | -3.47 | 0.40 | -16.72 | 10.00 | 22 |
|  | 2015 | -2.93 | 0.49 | -17.67 | 14.83 | 21 |
|  | 2016 | 0.31 | 0.37 | -11.22 | 16.83 | 1 |
|  | 2017 | -0.75 | 0.39 | -13.61 | 13.33 | 5 |
| Farmington | 2013 | -2.65 | 0.67 | -15.83 | 13.00 | 9 |
|  | 2014 | -4.51 | 0.79 | -19.44 | 9.66 | 29 |
|  | 2015 | -4.03 | 0.94 | -20.44 | 14.20 | 31 |
|  | 2016 | -0.96 | 0.76 | -20.00 | 16.90 | 8 |
|  | 2017 | -1.67 | 0.78 | -16.38 | 14.10 | 15 |
| Phelps | 2013 |  |  |  |  |  |
|  | 2014 |  |  |  |  |  |
|  | 2015 | -2.4 | 0.48 | -2.66 | 14.40 | 0 |
|  | 2016 | 2.37 | 0.72 | -19.70 | 16.10 | 5 |
|  | 2017 | -0.67 | 0.72 | -14.60 | 14.20 | 7 |
